# Analysis of clinical factors associated with incomplete standard protocol ^177^Lu-DOTATATE treatment in neuroendocrine tumor patients

**DOI:** 10.1007/s11604-025-01769-7

**Published:** 2025-03-29

**Authors:** Shigeyasu Sugawara, Shozo Okamoto, Hirofumi Go, Shiro Ishii, Hiroshi Ito, Tohru Shiga, Noboru Oriuchi

**Affiliations:** 1 Advanced Clinical Research Center, Fukushima Medical University, 1 Hikarigaoka, Fukushima, Fukushima Japan; 2 Department of Radiology and Nuclear Medicine, Fukushima Medical University, Fukushima, Japan

**Keywords:** ^177^Lu-DOTATATE, Neuroendocrine tumors (NETs), Peptide receptor radionuclide therapy (PRRT), Targeted radionuclide therapy (TRT)

## Abstract

**Purpose:**

To investigate the clinical features of patients with neuroendocrine tumors (NETs) who could not complete the standard protocol (4 cycles of 7.4 GBq per 8 weeks) ^177^Lu-DOTATATE treatment**.**

**Materials and methods:**

A retrospective single-center analysis was conducted on 26 patients who underwent ^177^Lu-DOTATATE treatment between December 2021 and August 2024. Therapeutic outcome was compared with clinical features, including location and number of metastatic lesions, interval from diagnosis to the first ^177^Lu-DOTATATE treatment, and laboratory data. Statistical analyses were performed to identify clinical features associated with dose reduction or treatment discontinuation.

**Results:**

The clinical data of 24 patients with metastatic neuroendocrine tumors (NETs) were analyzed, of whom 16 patients completed the standard protocol ^177^Lu-DOTATATE treatment. The most common adverse events were hematologic toxicities. Eight patients did not complete the standard protocol treatment, primarily due to adverse events (6/8). Single variable logistic regression analysis revealed that the presence of somatostatin receptor scintigraphy (SRS) positive bone metastases (OR = 21.0, 95% CI 2.37–186, *p* = 0.006) and lower hemoglobin levels (OR = 0.479, 95% CI 0.255–0.900, *p* = 0.022) were significantly associated with incomplete treatment. Notably, 5/8 patients in the incomplete group had extensive bone metastases (> 20 lesions), including 4 with diffuse metastases. Other variables, including age, sex, white blood cell count, platelet count, eGFR, and other metastatic sites, showed no significant association with treatment completion.

**Conclusions:**

In this study, the presence of SRS-positive bone metastases and low hemoglobin levels were significant factors associated with the inability to complete ^177^Lu-DOTATATE treatment for NET patients. Extensive bone metastases, such as diffuse metastasis or more than 20 bone metastases, may be particularly associated with the inability to administer standard protocol treatment.

## Introduction

Neuroendocrine tumors (NETs) are derived from various organs in the neuroendocrine system, including the pancreas and gastrointestinal tract. The first choice of treatment for NETs is surgery. In the case of metastases or recurrence, somatostatin analogs are used to control tumor growth because most NET expresses somatostatin receptors (SSTR) on their cell surfaces. Chemotherapy and molecular-targeted agents are used for treatment-resistant tumors. Radionuclide therapy with radiolabeled somatostatin analogs known as peptide receptor radionuclide therapy (PRRT), such as ^177^Lu-DOTATATE, shows favorable therapeutic effects and less toxicity [1]. The NETTER-1 trial is a representative clinical study that evaluated the efficacy and safety of ^177^Lu-DOTATATE in patients with advanced midgut neuroendocrine tumors. In this pivotal trial, ^177^Lu-DOTATATE demonstrated significantly longer progression-free survival (65.2% vs. 10.8% at 20 months) and higher response rates (18% vs 3%) than high-dose octreotide therapy in midgut NETs [1]. In the long-term follow-up of the NETTER-1 trial published in 2021, the median overall survival was 36.3 months (95% confidence interval 25.9–51.7) in the standard therapy alone group versus 48.0 months (95% confidence interval 37.4–55.2) in the ^177^Lu-DOTATATE plus standard therapy combination group [2]. Across studies, the objective response rates with the ^177^Lu-DOTATATE range from 16 to 37%, with an additional 41–66% of patients achieving stable disease. Median progression-free survival ranges from 16 to 36 months [3, 4].

^177^Lu-DOTATATE therapy has been available to treat somatostatin receptor-positive gastroenteropancreatic neuroendocrine tumors (GEP-NETs) in Japan, and our institution started the therapy in 2021. The standard treatment regimen (standard protocol) of PRRT involves four cycles of 7.4 GBq ^177^Lu-DOTATATE administered intravenously at intervals of 8 weeks (± 1 week) based on the protocol of the NETTER-1 trial [3]. Although ^177^Lu-DOTATATE treatment is safe and effective, as described above, there are occasional adverse events or rapid disease progression that make it difficult to complete treatment. Hematological toxicity is a significant concern with ^177^Lu-DOTATATE therapy. Several studies [1, 5–10] have shown hematological toxicity as a common adverse event. Most of them had mild hematological toxicity, but a portion of patients experienced hematological toxicity above grade 3, according to the CTCAE (ver. 3.0–5.0). Acute myeloid leukemia (AML), myelodysplastic syndromes (MDS), and nephrotoxicity were reported in a few cases. Other adverse events included nausea, vomiting, decreased appetite, fatigue, headache, and alopecia. In the NETTER-1 trial, out of the 103 patients who completed the treatment phase, 24 (23%) could not complete all 4 administrations of ^177^Lu-DOTATATE, and 8 required dose reduction [1]. However, factors accounting for discontinuation or dose reduction were not indicated in either the primary or final analysis of the NETTER-1 trial [1, 2]. We experienced a considerable number of cases in which patients could not complete standard protocol ^177^Lu-DOTATATE treatment because of adverse events or other reasons. Suppose we know which patients could not take the standard protocol ^177^Lu-DOTATATE. In that case, we can adjust the radioactivity dose and schedule in advance to make the treatment safer and more effective while still following the current protocol. This study aimed to investigate the clinical features of patients who could not complete the standard protocol ^177^Lu-DOTATATE treatment for NETs.

## Materials and methods

### Participants

This study was conducted after receiving approval from the Institutional Ethics Committee at Fukushima Medical University (REC2023-160). A total of 26 patients with metastatic NETs were assigned in the present study. NET diagnosis was confirmed by biopsy, laboratory examination, and imaging examination. They had undergone ^177^Lu-DOTATATE treatment at our institution between December 2021 and August 2024. All of them showed SSTR expression in lesions and had received at least one cycle of ^177^Lu-DOTATATE (3.7–7.4 GBq) treatment. We excluded patients who had already received at least one cycle of ^177^Lu-DOTATATE treatment at another institution and patients who received subsequent cycles of ^177^Lu-DOTATATE treatment at another institution.

### ^***177***^***Lu-DOTATATE treatment protocol***

Patients had undergone screening evaluation before ^177^Lu-DOTATATE treatment, which included contrast-enhanced computed tomography (CECT), somatostatin receptor scintigraphy (SRS), documentation of clinical symptoms, and laboratory evaluation (urinalysis, hematology, blood biochemistry) before the first administration of ^177^Lu-DOTATATE. Laboratory evaluation was conducted the day before or the same day as the ^177^Lu-DOTATATE administration and the follow-up evaluation. CECT imaging and laboratory evaluation was done typically three months after the last treatment cycle. Long-acting somatostatin analogs had been stopped four weeks before ^177^Lu-DOTATATE administration, while short-acting analogs can be continued until 24 h before the therapy. Intravenous injection of a mixed amino acid solution (25 g L-lysine hydrochloride and 25 g L-arginine hydrochloride in 1L normal saline) was started 30 min before ^177^Lu-DOTATATE administration and continued for 4 h. A standard dose of 7.4 GBq ^177^Lu-DOTATATE was administered every 8 weeks for a total of four doses. A reduced dose of 3.7 GBq was administered for patients with grade 2 or higher thrombocytopenia, Creatinine clearance (Ccr) < 40 mL/min, or other grade 3 or higher adverse events. Treatment was discontinued if these conditions did not improve within 16 weeks from the previous administration or if they recurred after dose reduction. The patients took Granisetron Hydrochloride (2 mg) one hour before ^177^Lu-DOTATATE administration. All treated patients were admitted to the radiation therapy room for each injection and stayed until the release criteria were fulfilled.

### Somatostatin receptor scintigraphy

All patients were confirmed of SSTR expression in the lesions by SRS within six months before ^177^Lu-DOTATATE treatment. Five of the 26 patients underwent SRS at our institution, while 21 were performed at other institutions. Those 21 patients did not undergo additional SRS in our institution to avoid further radiation exposure and cost. In our institution, SRS was performed after intravenous injection of 222 MBq ^111^In–labeled pentetreotide. At other institutions, a dose of 111–222 MBq of ^111^In–labeled pentetreotide was employed. Images were acquired 4 h and 27 h after administration. A whole-body scintigraphy scan was performed using a dual-head gamma camera (E.CAM, Cannon Co. Ltd., Tokyo, Japan) equipped with a parallel hole middle energy collimator with the patient lying supine on the imaging table. Anterior and posterior images were acquired into 1,024 × 256 matrices in a single pass at 10 cm per minute speed at 4 h after administration and 8 cm per minute speed at 27 h after administration. The evaluation of SRS used the Krenning score [11], and lesions of score 2 or more were counted as positive lesions.

### Evaluation of tumor response

Tumor response was classified according to RECIST 1.1 (Response Evaluation Criteria in Solid Tumors) with the follow-up CECT scan that had been done typically three months after the last treatment cycle [12].

### Toxicity assessment

^177^Lu-DOTATATE treatment-related toxicity was identified based on laboratory evaluation in screening and follow-up evaluation, documentation of clinical symptoms, and ECOG PS (Eastern Cooperative Oncology Group Performance Status) in clinic visits. All adverse events (AEs) were categorized according to the Common Terminology Criteria for Adverse Events (CTCAE), Version 5**.**

### Statistical analysis

The main objective of this study was to investigate the clinical features of patients with NETs who could not complete the standard protocol ^177^Lu-DOTATATE treatment. The presence of SRS-positive bone and lymph node metastases, the interval from diagnosis to the first ^177^Lu-DOTATATE treatment, age, sex, and laboratory data (The pretreatment number of hemoglobin (Hb) and platelet (Plt), white blood cell (WBC), estimated glomerular filtration rate (eGFR)) were statistically analyzed. All patients had liver metastases, so the analysis did not consider this factor. These variables for analysis were selected based on our clinical relevance and interest. These clinical features between the incomplete and complete groups were analyzed using single-variable logistic regression. A p-value of < 0.05 was considered statistically significant. R version 4.4.1 (The R Foundation for Statistical Computing, Vienna, Austria) was used for all analyses.

## Results

**Fig. 1:**
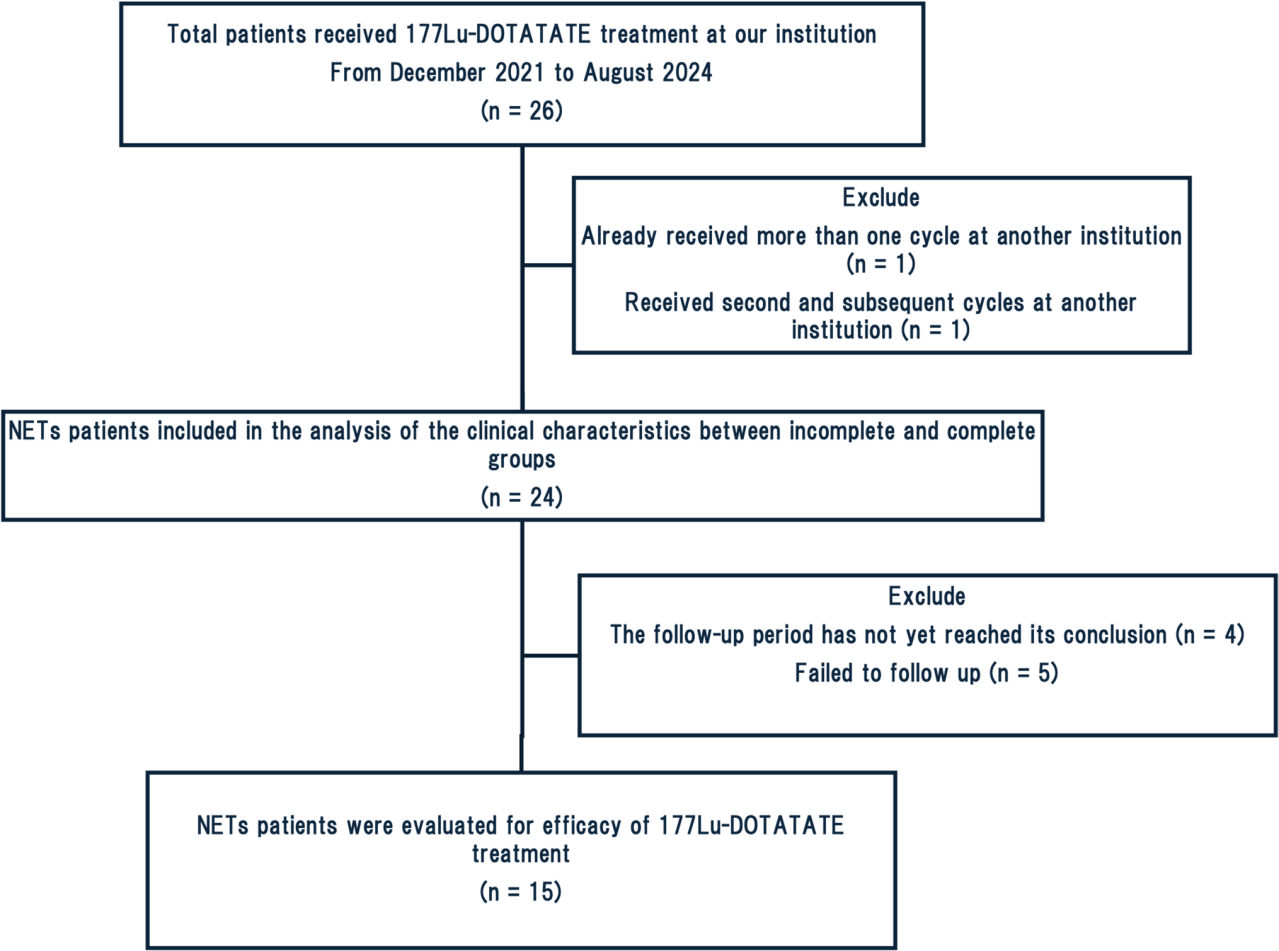
Patient flow diagram

Twenty-six patients with metastatic neuroendocrine tumors (NETs) underwent ^177^Lu-DOTATATE treatment. Two patients were excluded from this study because one had already received more than one cycle of ^177^Lu-DOTATATE treatment at another institution, and another received second and subsequent cycles of ^177^Lu-DOTATATE treatment at another institution (Fig. 1). A total of 24 patients (11 male and 13 female) were included in the analysis. Sixteen patients completed the standard protocol treatment. The results of SRS demonstrated the presence of radiotracer uptake in the metastatic lesions in all patients. All the patients had liver metastases. Bone metastases were observed in 8 patients (6 in the incomplete group and 2 in the complete group), and lymph node metastases were observed in 10 patients (one in the incomplete group and nine in the complete group). The demographic and clinical features of the patients are summarized in Table 1.

**Table 1:** Patient characteristics

Characteristic	Overall, *n* = 24	^177^Lu-DOTATATE treatment	
		Incomplete group, *n* = 8^a^	Complete group, *n* = 16^a^
Gender			
Female	14	4	10
Male	10	4	6
Age(years)	67.50 [57.75–70.25]	64.50 [59.00–70.75]	68.50 [57.75–70.25]
Primary lesion			
Duodenum	1	0	1
Ileocecum	1	1	0
Pancreas	8	3	5
Rectum	11	3	8
Renal	1	0	1
Unknown	2	1	1
Bone metastases			
Positive	8	6	2
Negative	16	2	14
Number of bone metastases			
0	16	1	15
1–19	3	1	2
20≦	5	5^b^	0
Liver metastases			
Positive	24	8	16
Negative	0	0	0
Lymph node metastases			
Positive	10	1	9
Negative	14	7	7
NET grade			
1	3	1	2
2	19	6	13
3	1	1	0
Unknown	1	0	1
Pre-Laboratory evaluation			
Creatinine (mg/dl)	0.70 [0.58–0.80]	0.72 [0.68–0.78]	0.70 [0.57–0.82]
eGFR (ml/min/l)	76.50 [66.50–87.25]	82.00 [61.50–91.00]	74.50 [69.25–86.50]
Hemoglobin (g/dl)	12.05 [10.50–13.93]	10.80 [9.55–11.72]	13.60 [11.72–14.15]
Platelet (10*3/μl)	205.50 [172.25–312.50]	201.50 [163.25–240.25]	205.50 [186.25–338.00]
White Blood Cell (10*3/μl)	4.70 [4.02–6.38]	3.85 [3.35–4.65]	5.55 [4.35–6.80]
Interval from diagnosis to the first PRRT (months)	57.50 [20.00–133.50]	32.50 [21.25–116.00]	68.50 [20.00–145.25]

^a^Statistics presented: median [IQR]; *n*

^b^Four cases with diffuse bone metastases were included

Table 2 describes AEs during and after treatment. The most common AE was hematologic toxicity, including leukopenia in 13 patients, anemia in 14 patients, and thrombocytopenia in 11 patients. Among them, grade 3 and 4 adverse events were leukopenia in 4 patients, anemia in three, and thrombocytopenia in two. Other grade 3 and 4 AEs were diarrhea, acute kidney injury, and hypokalemia. These AEs were found in the same patient with symptoms of watery diarrhea, hypokalemia, and achlorhydria syndrome due to a vasoactive intestinal polypeptide-producing tumor. Of the 24 patients, 8 (4 male and 4 female) did not complete the standard protocol ^177^Lu-DOTATATE treatment. AEs were the primary reason for discontinuation in 6 patients, disease progression in one patient, and social factors in the other.

**Table 2:** Adverse events summary

Adverse event	Number (%)	
	Overall^a^	Grade 3–4^a^
Leukopenia	13	4
Anemia	14	3
Thrombocytopenia	11	2
nausea	3	
constipation	1	
pain	4	
Anorexia	4	
Fatigue	2	
Dizziness	1	
Diarrhea^b^	1	1
Acute Kidney Injury^b^	1	1
Hypokalemia^b^	1	1

^a^Statistics presented: *n*

^b^These AEs were found in the same patients as symptoms of WDHA syndrome

A single variable logistic regression was conducted to identify factors associated with incomplete treatment with ^177^Lu-DOTATATE. The presence of bone metastases was significantly associated with a higher likelihood of incomplete treatment (OR = 21.0, 95% CI 2.37–186, *p* = 0.006). The analysis revealed that patients with bone metastases were more likely to receive reduced doses or experience treatment discontinuation. Among those in the incomplete treatment group, 5 patients presented with more than 20 bone metastases, and 4 of these had diffuse bone metastases. In contrast, no patients in the complete treatment group had more than 20 bone metastases. Additionally, incomplete treatment was significantly associated with lower hemoglobin levels (OR = 0.479, 95% CI 0.255–0.900, *p* = 0.022). This means that the probability of incomplete treatment is reduced by around 52% with every unit rise in hemoglobin. The presence of lymph node metastases (OR = 0.111, 95% CI 0.011–1.130, *p* = 0.063), the interval between diagnosis to the first ^177^Lu-DOTATATE treatment (OR = 0.997, 95% CI 0.984–1.010, *p* = 0.596), eGFR (OR = 1.01, 95% CI 0.965–1.050, *p* = 0.778), and age (OR = 0.995, 95% CI 0.913–1.090, *p* = 0.918), platelet count (OR = 0.998, 95% CI 0.992–1.000, *p* = 0.583), and white blood cell count (OR = 0.851, 95% CI 0.586–1.240, *p* = 0.397) did not show significant associations with the incomplete or complete of ^177^Lu-DOTATATE treatment (Table 3).

**Table 3:** Associations of incomplete ^177^Lu-DOTATATE treatment

		Odds Ratio	95% confidence interval			*p* value
Sex	Male	1.29	0.234	–	7.050	0.772
	Female	Reference				
Age		0.995	0.913	–	1.090	0.918
Hb		0.479	0.255	–	0.900	0.022
Plt		0.998	0.992	–	1.000	0.583
WBC		0.851	0.586	–	1.240	0.397
Bone metastases	Positive	21.0	2.37	–	186	0.006
	Negative	Reference				
Lymph node metastases	Positive	0.111	0.011	–	1.130	0.063
	Negative	Reference				
Interval from diagnosis to the first ^177^Lu-DOTATATE treatment		0.997	0.984	–	1.010	0.596
eGFR		1.010	0.965	–	1.050	0.780

Odds ratios were calculated using single-variable logistic regression analysis. Values of *p* < 0.05 were considered statistically significant

Response to therapy was assessed in 15 patients. A total of 24 patients were included in the analysis of clinical features between incomplete and complete groups. However, 9 patients were excluded from the final analysis. Four patients were excluded due to incomplete three-month follow-up surveys, while 5 patients were excluded due to loss of follow-up. One patient had progressive disease, 1 partial response (PR), and 3 stable disease (SD) in the incomplete groups, while 6 showed PR and 4 SD in the complete groups. There were no significant differences in the response between these two groups (*p* = 0.320).

## Discussion

**Fig. 2:**
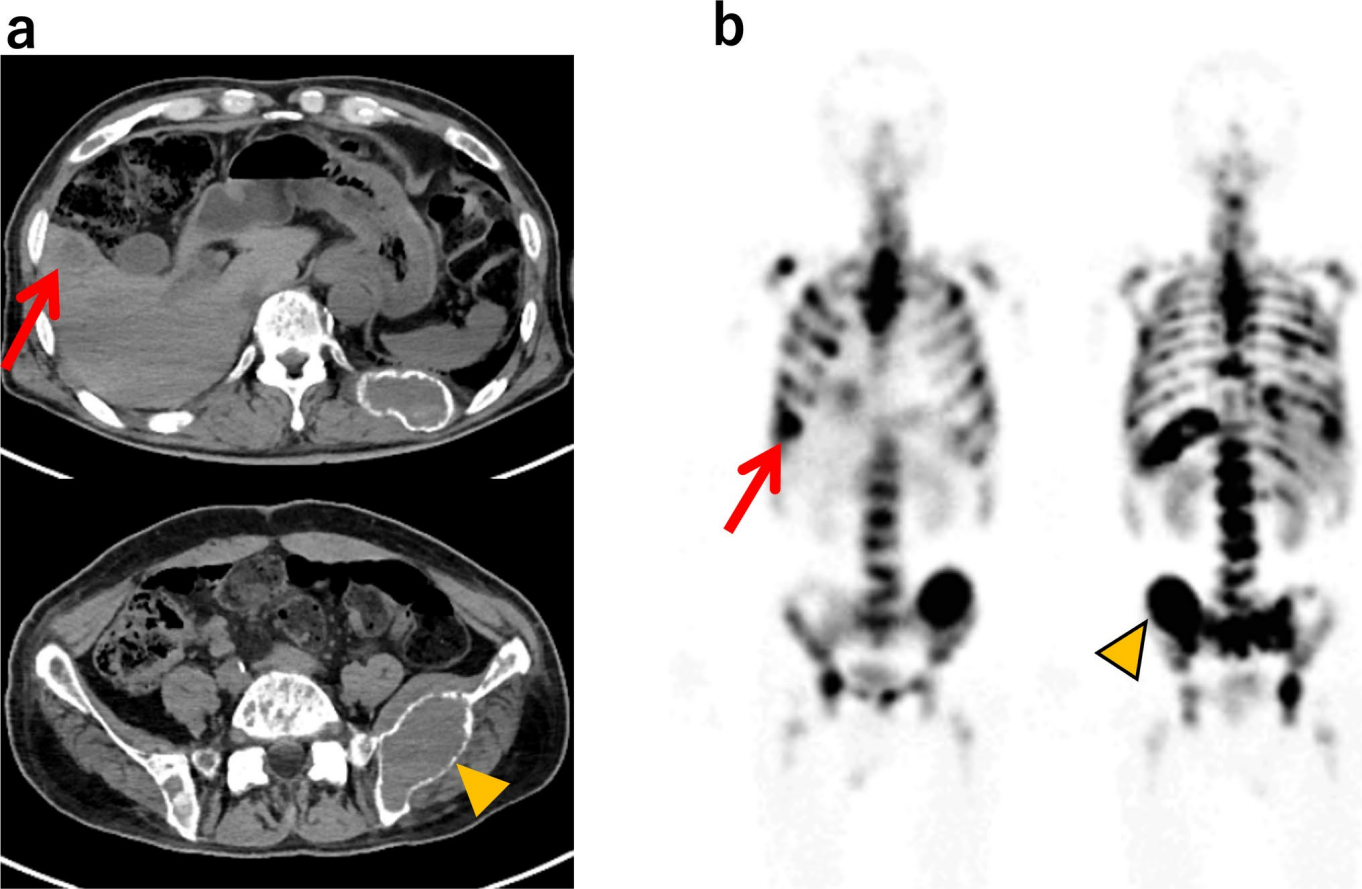
A case of incomplete ^177^Lu-DOTATATE treatment. **a** CT axial view, **b** ^177^Lu-DOTATATE scintigraphy 27 h after the first dose. The patient was a 65-year-old male with liver metastases (arrow) and diffuse bone metastases (arrowhead). After the first administration of ^177^Lu-DOTATATE, hemoglobin (Hb) and platelet (Plt) decreased to Grade 3 in CTCAE 5.0. The second and subsequent administrations could not be performed because Hb and Plt did not recover by 16 weeks after the first administration

The study identified two main clinical determinants associated with incomplete standard protocol ^177^Lu-DOTATATE treatment in patients with NETs. These were the presence of bone metastases and lower pretreatment hemoglobin levels. To add to this association, we calculated the odds ratio for the presence of bone metastases. A closer examination of the incomplete group revealed a predominance of cases with extensive bone metastases, such as diffuse metastasis or more than 20 bone metastases. This observation suggested that more bone metastases might be associated with a decreased likelihood of completing the treatment regimen. The radiation dose to the bone marrow was expected to be elevated in the event of multiple bone metastases, as shown in the ^177^Lu-DOTATATE scintigraphy (Fig. 2). In patients with multiple bone metastases, hematological adverse effects (AEs) with the administration of ^177^Lu-DOTATATE might occur due to reduced bone marrow reserve. Therefore, it can be necessary to reduce the dosage or withhold treatment. These findings indicate that the extent of bone metastasis and baseline hematologic status are crucial factors influencing the feasibility of the current therapeutic protocol. Dose modification for ^177^Lu-DOTATATE therapy has been examined to enhance efficacy compared to the NETTER-1 trial that included only induction therapy with a fixed dose [12]. The study applied dose adjustment according to the clinical features, such as renal function extent of bone and liver metastases, and previous therapy, and added low-dose maintenance therapy to the four administrations of induction therapy to be an efficacious therapeutic strategy in various types of metastatic NETs [13]. Considering these findings, the incomplete group in our study might improve the tolerability and efficacy by a prior dose reduction with the addition of maintenance therapy to increase the overall dosage. Increased dosage could enhance the therapeutic efficacy of the ^177^Lu-DOTATATE treatment. The results demonstrated no significant difference in the efficacy between the two groups in the present study. However, there was a trend toward a higher PR rate in the complete group. These results were due to the small sample size and the evaluation limitations resulting from the insufficient follow-up period. Furthermore, a considerable proportion (37.5%) of patients in the incomplete group were not subjected to follow-up assessments.

This study has several important limitations. First, we could not perform multivariate logistic regression analysis as a retrospective single-center study with a relatively small sample size (*n* = 24) and particularly few patients in the incomplete treatment group (*n* = 8). Consequently, potential confounding factors were not fully accounted for, and the results lack causal inference. And a multicenter study incorporating a larger number of cases would be necessary to conduct more accurate assessments. Furthermore, factors that affect the completion of the standard protocol treatment might not be identified beyond bone metastasis and hemoglobin level. Additionally, as mentioned above, the statistical analysis of tumor response for the ^177^Lu-DOTATATE treatment was insufficient. A more extensive population study could clarify these issues. Second, the quality of pretreatment SRS images might be inconsistent because they were performed at different institutions. Although all imaging should have been performed at the same institution, this was impossible because many patients lived far away, and imaging at our institution would have been inconvenient. These images were evaluated by 2 professional radiologists, who confirmed that there were no significant issues with image quality. However, these variations may have influenced the interpretation of bone metastasis findings, potentially leading to variability in diagnostic accuracy and reliability across different cases. To mitigate this issue, future studies should aim to standardize imaging protocols across institutions. Third, it is not ideal that the number of positive lesions on SRS images evaluated the bone metastases. The utility of quantitative indices such as the bone scan index (BSI) has been used to assess osteogenic bone metastasis in patients with prostate cancer, as well as other tumor types; however, bone scintigraphy was not performed in most patients in this study [13]. Since no other quantitative evaluation methods, including the BSI, have been established for SRS, this study visually assessed the number of bone metastases as an alternative evaluation method. Despite these limitations, our study provides valuable insights into the clinical utility of SRS imaging for assessing bone metastases.

## Conclusion

In this study, SRS-positive bone metastases and low hemoglobin levels were significant factors associated with the inability to complete ^177^Lu-DOTATATE treatment for NET patients. Extensive bone metastases, such as diffuse metastasis or more than 20 bone metastases, may be particularly associated with the inability to administer standard protocol treatment. Therefore, ^177^Lu-DOTATATE treatment for those patients should be planned carefully to ensure its completion safely and effectively.
